# Do bisphosphonates affect bone healing? A meta-analysis of randomized controlled trials

**DOI:** 10.1186/1749-799X-9-45

**Published:** 2014-06-05

**Authors:** Deting Xue, Fangcai Li, Gang Chen, Shigui Yan, Zhijun Pan

**Affiliations:** 1 Department of Orthopaedics, 2nd Affiliated Hospital, School of Medicine, Zhejiang University, #88 Jiefang Road, Hangzhou 310009, China

**Keywords:** Bisphosphonates, Indirect bone healing, Lumbar fusion, Randomized controlled trials, Meta-analysis

## Abstract

**Background:**

Whether bisphosphonates affect indirect bone healing is still unclear.

**Method:**

We carried out a comprehensive search strategy. Only randomized controlled trials were included. Two reviewers independently assessed methodological qualities and extracted outcome data. Analysis was performed with RevMan 5.2.

**Results:**

Eight eligible randomized controlled trials with 2,508 patients were included. Meta-analysis results showed that no statistically significant differences were founded in indirect bone healing in short time (within 3 months) (relative risk (RR) 1.40, relative the control group; 95% CI 0.36 to 5.49) and in long-term (more than 12 months) postoperation (RR 1.0; 95% CI 0.98 to 1.02) between bisphosphonates infusion groups and control groups. There were no statistically significant differences of indirect bone healing between early and delay bisphosphonates administration groups. Bisphosphonates infusion after lumbar infusion surgery could promote bone healing and shorten fusion time in 6 months postoperation (RR 1.35; 95% CI 1.11 to 1.66).

**Conclusion:**

There was no clinically detectable delay to fracture healing via external callus formation following bisphosphonates treatment. Considering the benefit aspects of bisphosphonates for osteoporosis treatment, we recommend bisphosphonates infusion after fracture fixation surgery and lumbar fusion surgery.

## Introduction

There are two kinds of drugs which affect bone remolding, anabolic and anti-catabolic drugs. Anabolic drugs influence the osteoblasts and increase osteogenesis [1]. However, the high costs of anabolic drugs limit their wide applications. So most countries recommend anti-catabolic drugs as the first line drugs for treating osteoporosis, especially bisphosphonates [2]. Bisphosphonates have an inhibitory effect on osteoclastic bone resorption. So they have been used in diseases with increased bone turnover, such as osteoporosis, Paget's disease, and metastatic bone diseases [3-5]. In theory, bisphosphonates inhibit osteoclast-mediated bone resorption which would prevent bone loss and improve bone strength [6,7]. But treating patients with bisphosphonates during bone healing is controversial because osteoclasts are important for remodeling callus into cortical bone [8]. Several studies have addressed the effects of bisphosphonates on indirect bone healing. The results are inconsistent. Lenehan et al. [4] found that ethane-1-hydroxy-1,1-diphosphonate inhibited indirect fracture healing in a dose-dependent manner in mature beagle dogs. In a prospective study, Kim et al. found that bisphosphonates did not affect intertrochanteric indirect fracture healing [3]. A randomized study of 32 postmenopausal women with Colles' fractures showed that the clodronate increased mineralization of callus [5]. So shall we use bisphosphonates for the patients with osteoporotic fractures or spinal fusion?

In order to summarize available randomized control trials and make this issue clear, we performed a meta-analysis of available randomized evidence to evaluate whether bisphosphonates affect bone healing.

## Methods and materials

We searched Medline (1966–May 2013), Embase (1980–May 2013), Science Citation Index (1981–May 2013), Cochrane Central Register of Controlled Trials (CENTRAL), and Cochrane Database of Systematic Reviews (Cochrane Library, Issue 1, 2013) for randomized clinical trials that evaluated the effect of bisphosphonates on bone healing. We also searched unpublished trials and those in progress using clinical trials repositories, including the National Institute of Health (May 2013), the National Research Register (May 2013), and Current Controlled Trials (May 2013). The following terms were used: ‘bisphosphonates’, ‘indirect bone healing,’ and ‘bone formation’. Searches were not restricted by year of publication or language. Reference lists of all included studies were scanned to identify additional potentially relevant studies. Two reviewers independently screened the titles and abstracts of identified papers, and full text copies of all potentially relevant studies were obtained.

### Study selection and outcomes

We included studies if they were randomized trials of bisphosphonates affecting fracture healing, regardless of the doses and duration of drugs used. No language restrictions were applied. We excluded studies that were not peer-reviewed randomized controlled trials, studies included patients with diseases that affecting bone mineral density (BMD) or bone metabolism, such as renal or adrenal insufficiency, diabetes, and any history of taking any medication known to affect BMD, such as corticosteroids.

Bone healing criteria: all of the study used radiographic examination to evaluate bone healing, but one study [5] did not provide bone healing criteria. The detailed criteria were shown in Table 1.

**Table 1:** Characteristics of included studies

**Study (country) years**	**Gender (M/F)**	**Number of patients (bisphosphonate/control)**	**Mean age (years)**	**Selection criteria**	**Intervention (calcium and/or vitamin D)**	**Time of starting treatment**	**Fracture healing criteria**	**Outcome measured**
van der Poest et al. (Netherlands) 2000 [7]	F	18/17	65.7	Distal forearm fractures, T-score ≤ 2	10 mg alendronate + 500 mg calcium vs. placebo + 500 mg calcium	2–4 weeks after fracture	Radiographs and functional outcome	BMD, bone markers, fracture healing
Adolphson et al. (Sweden) 2000 [5]	F	16/16	62	Displaced Colles' fracture; postmenopausal women	400 mg clodronate vs. placebo	48 h after fracture	Did not provide	BMD, function
Harding et al. (Sweden) 2011 [14]	M and F	25/21	48.9	Patients with osteoarthritis or deformity of knee; age between 35–65 years old	4 mg zoledronic acid vs. 9 mg/mL sodium chloride	4 weeks postoperation	Consolidation of about two-thirds of the osteotomy gap by radiographic examination	Time to healing; BMD
Nagahama et al. (Japan) 2011 [12]	M and F	19/17	68.9	Candidated for single-level PLIF; BMD was less than 70% of the young adult mean value	35 mg/week alendronate vs. 1 μg/day alfacalcidol	Just postoperation	Bridging bone formation through the cage by CT scan	BMD, bone union
Colon-Emeric et al. (USA, Norway, Belgium, Canada) 2011 [13]	M and F	1,065/1,062	Not reported	Patients within 90 days of hip fracture repair	5 mg zoledronic acid vs. placebo	Within 90 days postoperation	Clinical symptoms: without persistent pain, bear weight Radiographic examination	Fracture healing
Kim et al. (Korea) 2012 [3]	M and F	90/0	76.1	Patients with intertrochanteric fracture; BMD < −2.5	35 mg/week risedronate + 1,200 mg/day calcium + 800 IU/day cholecalciferol	2 weeks vs. 1 month vs. 3 months after operation	At least two cortices bridging at the fracture site	Fracture healing, function
Gong et al. (Korea) 2012 [15]	F	60/0	66.5	Distal radial fractures treated with plate fixation; BMD < −2.5	70 mg/week alendronate	2 weeks vs. 3 months after operation	Bridging by trabeculae or osseous bone in at least one cortex as seen on anteroposterior radiographs and one as seen on lateral radiographs	Fracture healing, function; complication
Li et al. (China) 2012 [16]	M and F	41/41	63.73	Patients undergoing TLIF	5 mg zoledronic acid + calcium 1000 mg/day + vitamin D 400 IU/day vs. placebo + calcium 1000 mg/day + vitamin D 400 IU/day	3 days postoperation	Bridging bony trabeculation through the cage by CT scan	Interbody fusion rate; function; biochemical assessment

We defined that evaluated indirect bone healing within 3 months as short time and more than 6 months as long term.

Delayed fracture union was defined as one or more clinical symptoms (pain, inability to ambulate, and gait disorder) at least 6 weeks after surgical repair plus radiographic findings. Fracture nonunion was defined as incomplete bony bridging through cages in lumbar spine in 12 months postoperation or no cortices bridging at the fracture site in more than 8 months postoperation.

The primary outcome was the indirect bone healing time and fracture nonunion.

### Data extraction

Two reviewers independently extracted the data information of trial characteristics, patient data, outcome measures, and study quality using a standardized protocol and reporting document. Disagreements were resolved by consensus. To quantify the level of agreement between reviewers, a *κ* statistic was calculated. The *κ* statistic is a chance-corrected proportional index, with values ranging from +1 (perfect agreement) to −1 (complete disagreement). Information extracted included personal information, methodology, details on interventions, and reported outcomes.

### Study quality assessment

We assessed the methods of every study according to Cochrane Handbook for Systematic Reviews of Interventions, including reporting of randomization method, allocation concealment, blinding of outcome assessment, and completeness of follow-up.

### Statistical analysis

The meta-analysis was done according to the Cochrane Collaboration and the Quality of Reporting of Meta-analyses guidelines (QUOROM) [9] with standard software (Review manager (RevMan), version 5.2, Cochrane collaboration) [10]. Heterogeneity was assessed with *I*^2^ statistics [11]. *I*^2^ is the proportion of total variation observed between the trials attributable to differences between trials rather than sampling error (chance). Relative risk (RR) was used as the summary statistic to perform statistical analysis of dichotomous variables. A fixed effect model was used for calculations of summary estimates and their 95% CI. However, when the heterogeneity was significant, a random effects model was used.

## Results

**Figure 1:**
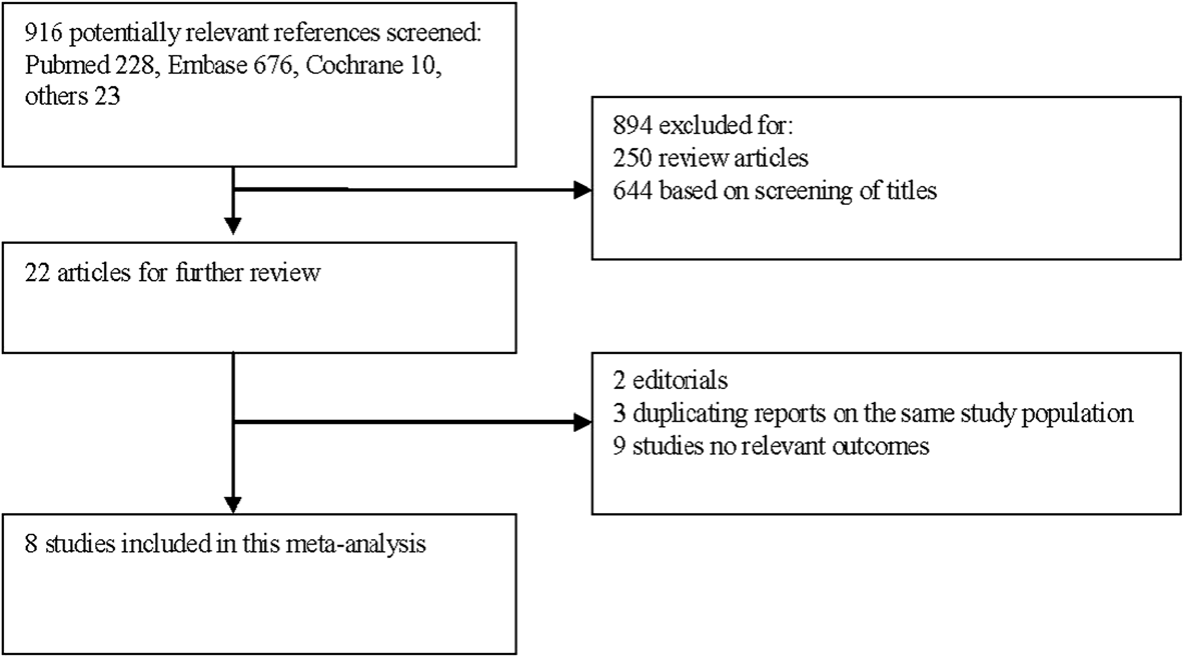
Selection of study.

We identified 916 potentially relevant publications. Eight trials [3,5,7,12-16] met the predefined inclusion criteria and were included in our meta-analysis (Figure 1). The eight trails included 2,508 patients (Table 1). Calcium supplement was used in three trials [3,7,16]. Three trials [5,7,15] included patients with distal radial fractures; two trials [3,13] included patients with posterior lumbar interbody fusion. Two trials [13,15] included patients with hip fracture; one trial [14] included patients with tibial osteotomy.

Six trials [5,7,12-15] compared bisphosphonate with placebo for indirect bone healing; two trials [3,15] compared different timing of postoperative administrating bisphosphonate on indirect bone healing.

After adjustment for the agreement between reviewers, the *κ* coefficient on the agreement of the included studies was 0.91 (95% confidence interval 0.82 to 0.94), suggesting good agreement between reviewers in data extraction. On the basis of quality assessment, five trials were deemed to be at low risks of bias and the remainder to be at high risks (Table 2).

**Table 2:** Quality assessment of included studies

**Study**	**Randomization**	**Allocated concealment**	**Blinding**	**Length of follow-up**	**Withdrawal/lost to follow-up (%)**
van der Poest et al. [7]	Unclear	Unclear	Unclear	12 months	10.8
Adolphson et al. [5]	Adequate	Adequate	Adequate	12 months	6
Harding et al. [14]	Adequate	Adequate	Adequate	18 months	0
Nagahama et al. [12]	Adequate	Adequate	Adequate	12 months	0
Colon-Emeric et al. [13]	Adequate	Adequate	Adequate	12 months	3.0
Kim et al. [3]	Adequate	Unclear	Unclear	12 months	0
Gong et al. [15]	Adequate	No	No	6 months	0
Li et al. [16]	Adequate	Adequate	Adequate	12 months	0

### Evaluation of bone healing in short term (within 3 months)

**Figure 2:**
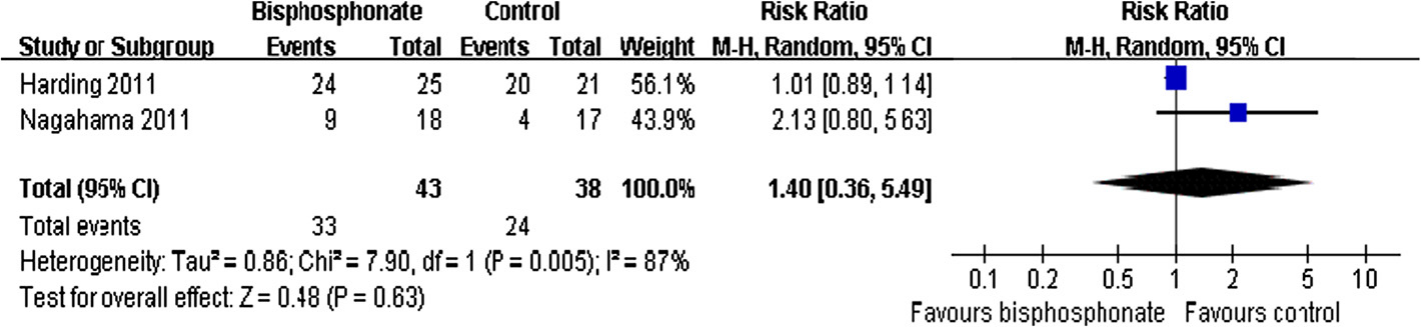
Evaluation of bone healing in short term.

This result was based on two studies [12,14] (138 participants). Because the examinations of bone healing at 3 months postoperation were heterogeneous among the included trials, we used a random effects model. The pooled RR was 1.40; 95% CI (0.36, 5.49). This result was not statistically significant (*P* = 0.63) (Figure 2).

### Evaluation of bone healing in long term (more than 6 months)

**Figure 3:**
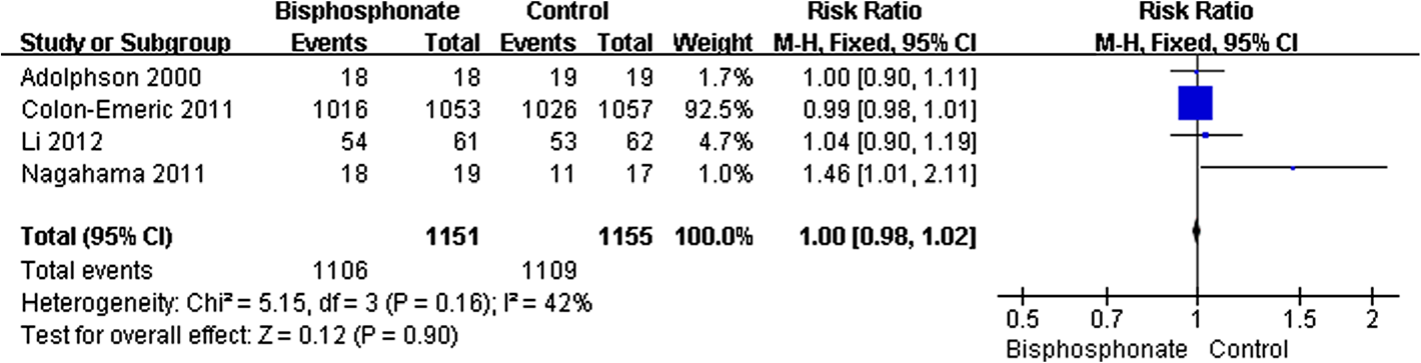
Evaluation of bone healing in long term.

This result was based on four studies [5,12,13,16] (2,306 participants). The test for homogeneity showed that results were consistent across trials (*P* = 0.16, *I*^2^ = 42%). The pooled RR was 1.0; 95% CI (0.98, 1.02). This result was not statistically significant (*P* = 0.90) (Figure 3).

### Delay bone healing or nonunion of fractures between administrating bisphosphonate groups and control groups

**Figure 4:**
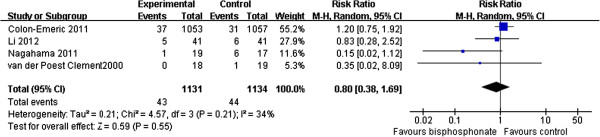
Delay bone healing or nonunion of fractures between administrating bisphosphonate groups and control groups.

Four trials [7,12,13,16] reported delay bone healing or nonunion of fractures (2,265 participants). The test for homogeneity showed that results were consistent across trials (*P* = 0.21, *I*^2^ = 34%). The pooled RR was 0.8; 95% CI (0.38, 1.69). This result was not statistically significant (*P* = 0.55) (Figure 4).

### The timing of zoledronic acid infusion on bone healing

**Figure 5:**
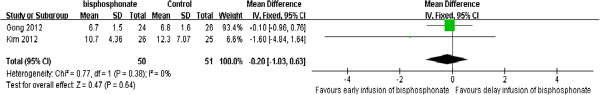
Evaluation of early and delay zoledronic acid infusion on bone healing.

Two trials [3,15] compared early (within 1 month of postoperation) and delay (after 1 month of postoperation) zoledronic acid infusion on bone healing. The test for homogeneity showed that results were consistent across trials (*P* = 0.38, *I*^2^ = 0%). Meta-analysis showed that the pooled RR was −0.20; 95% CI (−1.03, 0.63). This result was not statistically significant (*P* = 0.64) (Figure 5).

### Evaluation of bisphosphonate on lumbar fusion

Two studies [12,17] reported zoledronic acid infusion on lumbar fusion. The test for homogeneity showed that results were consistent across trials (*P* = 0.54, *I*^2^ = 0%) in 6 months postoperation but inconsistent across trials (*P* = 0.11, *I*^2^ = 61%) at 12 months postoperation. Meta-analysis showed that the pooled RR was 1.35; 95% CI (1.11, 1.66) at 6 months postoperation. This result was statistically significant (*P* = 0.003). But the pooled RR was 2.80; 95% CI (0.41, 19.24) in 12 months postoperation. This result was not statistically significant (*P* = 0.30).

## Discussion

The main objective of this meta-analysis was to assess whether bisphosphonates affect indirect bone healing. Because in lumbar fusion surgery, bone formation occurs via intramembranous ossification due to the relatively stable construct supporting the fusion site, so our analysis included spinal fusion. Meta-analysis results showed that no statistically significant differences were found in short- and long-term postoperation between two groups. The delay indirect bone healing or fracture nonunion was also not significant differences between two groups. The timing of bisphosphonates infusion did not affect indirect bone healing. In subgroup analysis, we found that bisphosphonate-treated groups had statistically significant higher lumbar infusion rate at 6 months postoperation.

It is still unclear whether bisphosphonates therapy affects indirect bone healing. Several animal studies have demonstrated that bisphosphonates treatment after surgery would increase callus formation, delay callus remodeling, but without influencing the strength of healed bone or bone healing [16-18]. Van der Poest [7] evaluated whether alendronate prevented bone loss in distal radius after Colles' fractures. The BMD of distal radius increased significantly at 3 and 6 months compared with that of the control group. There were no significant differences of anatomic and functional outcomes between the alendronate group and control group after 1 year follow-up. In a high tibial osteotomy study, Harding et al. [14] found that infusion of zoledronic acid increased the pin fixation of external fixation but did not affect the bone healing. Bisphosphonates reduced osteoclast activity, but more and more clinical results showed that they did not have advert effects on bone healing.

The timing of infusion bisphosphonates was a controversy. In an animal study, Amanat et al. [19] found that delayed infusion of zoledronic acid increased more callus volume compared to both saline and infusion of zoledronic acid immediately at the time of the fracture. However, our analysis results found that the timing of infusion bisphosphonate did not affect fracture healing. This was consistent with Colon-Emeric's report [13]. One possible reason may be that both included studies involved cancellous bone fractures. The spacious environment for cancellous new bone formation is large enough, so the bone remodeling process which suppressed by a reduction in the resorption process by bisphosphonate was not so important [15]. But the compact bones are different. Fracture bone debris needs to be absorbed to allow space for new bone formation [20]. Another reason may be that the dose of bisphosphonates for treating osteoporosis is sufficient for affecting bone healing in animal but insufficient for human.

It is interesting that bisphosphonates affect lumbar fusion in 6 months after operation in our study. The consistency across both studies was very well (*I*^2^ = 0%). This may be due to the enough spacious environment for osteogenesis without absorption process after decompression of the intervetebral discs. So bisphosphonates may have particular advantages for shortening intervetebral fusion in osteoporosis patients.

Our meta-analysis also showed that bisphosphonates did not delay indirect bone healing. Several studies have shown that the existence of a previous osteoporotic fracture conferred an approximately twofold higher risks of subsequent osteoporotic fracture [21]. So more and more authors recommended pharmacological intervention after first osteoporotic fracture to increase BMD and reduce the risks of subsequent fractures [22,23]. Health Outcomes and Reduced Incidence with Zoledronic Acid Once Yearly Recurrent Fracture Trial (HORIZON) showed that administration of zoledronic acid to patients with low-energy hip fractures would reduce the risk of subsequent vertebral or nonvertebral fractures [24].

Our meta-analysis had several limitations. Firstly, our results are subject to limitations inherent to any meta-analysis based on pooling of data from different trials with different doses of drugs, duration, fracture sites, and patient groups. Secondly, the reporting may be influenced by expectations of the investigators and patients. The surgical technique of doctors may also influence the healing results. Finally, the number of the included studies and participants were relatively small.

## Conclusions

In summary, the available data suggests that bisphosphonates do not cause a clinically detectable delay to indirect bone healing regardless of the timing of bisphosphonate delivery in relation to the fracture. However, the clinical studies reviewed are likely to be underpowered.

## Competing interests

The authors declare that they have no competing interests.

## Authors' contributions

The design of the study and preparation of the manuscript were done by DX and ZP. DX, FL, and GC assisted in the study processes, data collections, and preparations. SY and ZP assisted in the manuscript preparation. All authors read and approved the final manuscript.
